# The Employment of the Surface Plasmon Resonance (SPR) Microscopy Sensor for the Detection of Individual Extracellular Vesicles and Non-Biological Nanoparticles

**DOI:** 10.3390/bios13040472

**Published:** 2023-04-12

**Authors:** Nour Sharar, Konstantin Wüstefeld, Rahat Morad Talukder, Julija Skolnik, Katharina Kaufmann, Bernd Giebel, Verena Börger, Friedrich Nolte, Carsten Watzl, Frank Weichert, Roland Hergenröder, Victoria Shpacovitch

**Affiliations:** 1 Cell Therapy Center, The University of Jordan, Amman 11942, Jordan; n.sharar@ju.edu.jo; 2 Leibniz Institut für Analytische Wissenschaften-ISAS-e.V., Bunsen-Kirchhoff Straße 11, 44139 Dortmund, Germany; rahat.talukder@ruhr-uni-bochum.de (R.M.T.); julija.skolnik@isas.de (J.S.); katharina2.kaufmann@tu-dortmund.de (K.K.); roland.hergenroeder@isas.de (R.H.); 3 Department of Computer Science, TU Dortmund University, 44227 Dortmund, Germany; konstantin.wuestefeld@tu-dortmund.de (K.W.); frank.weichert@tu-dortmund.de (F.W.); 4 Institute for Transfusion Medicine, University Clinic Essen, 45122 Essen, Germany; bernd.giebel@uk-essen.de (B.G.); verena.boerger@uk-essen.de (V.B.); 5 University Medical Center Hamburg-Eppendorf, Institute of Immunology, 20246 Hamburg, Germany; nolte@uke.de; 6 Leibniz Research Centre for Working Environmental and Human Factors (IfADo), 44139 Dortmund, Germany; watzl@ifado.de

**Keywords:** surface plasmon resonance (SPR), SPR microscopy, extracellular vesicles, viruses, virus-like particles, sensor imaging, deep learning, imaging artifacts, sensor–actuator coupling

## Abstract

A wide-field surface plasmon resonance (SPR) microscopy sensor employs the surface plasmon resonance phenomenon to detect individual biological and non-biological nanoparticles. This sensor enables the detection, sizing, and quantification of biological nanoparticles (bioNPs), such as extracellular vesicles (EVs), viruses, and virus-like particles. The selectivity of bioNP detection does not require biological particle labeling, and it is achieved via the functionalization of the gold sensor surface by target-bioNP-specific antibodies. In the current work, we demonstrate the ability of SPR microscopy sensors to detect, simultaneously, silica NPs that differ by four times in size. Employed silica particles are close in their refractive index to bioNPs. The literature reports the ability of SPR microscopy sensors to detect the binding of lymphocytes (around 10 μm objects) to the sensor surface. Taken together, our findings and the results reported in the literature indicate the power of SPR microscopy sensors to detect bioNPs that differ by at least two orders in size. Modifications of the optical sensor scheme, such as mounting a concave lens, help to achieve homogeneous illumination of a gold sensor chip surface. In the current work, we also characterize the improved magnification factor of the modified SPR instrument. We evaluate the effectiveness of the modified and the primary version of the SPR microscopy sensors in detecting EVs isolated via different approaches. In addition, we demonstrate the possibility of employing translation and rotation stepper motors for precise adjustments of the positions of sensor optical elements—prism and objective—in the primary version of the SPR microscopy sensor instrument, and we present an algorithm to establish effective sensor–actuator coupling.

## 1. Introduction

Surface plasmon resonance (SPR) microscopy sensors are highly sensitive and flexible instruments [1,2]. Most conventional commercial SPR-based biosensors harness the relatively simple Kretschmann’s [3] scheme of plasmon excitation to detect target biological substances in liquid samples [4]. The simplicity of this optical scheme, and the high sensitivity of SPR sensors, have led to the employment of such instruments across the broad spectrum of biological applications: measurements of binding constants and kinetics of biomolecular interactions, concentration measurements, and many others; however, each sensor using this scheme has its merits and limitations [5]. For a long time, despite their high sensitivity, SPR-based sensors were not considered appropriate instruments for detecting individual bioNPs in liquid samples. Recently, results from independent research teams have proved the applicability of SPR-based sensors to the detection and quantification of individual bioNPs [6,7,8,9,10].

Considered an analytical instrument suitable for the real-time sizing and quantification of individual viruses and virus-like particles (VLPs) [7,11,12], the SPR microscopy sensor has also demonstrated its features by analyzing samples containing microvesicles–a subgroup of extracellular vesicles [6]. SPR microscopy enables the specific detection of individual bioNPs in liquid samples without needing biological labeling [2,5] and can directly compare relative concentrations of microvesicles in such samples [6].

Similarly to rapid antigen tests, an antigen-specific antibody coating on the gold sensor surface provides biological selectivity against a specific virus or extracellular vesicle [2]. Additional selectivity against the unspecific background is facilitated by the sizing capabilities [6] and temporal profiles of the binding event recognized by a detection algorithm [13].

Different materials have been employed as sensor surfaces, potentially improving optical performance [5,14,15]. Some of the materials employed in SPR-based sensors have facilitated the coupling of the plasmonic technique to other well-known techniques, such as fluorescent spectroscopy [16,17]. However, gold as a surface is chemically inert, stable against oxidation under atmospheric or liquid conditions, and can be easily produced and cleaned. Moreover, surface functionalization with biologically relevant molecules is well-established for gold films. Therefore, from the practical employment point of view, gold has clear benefits over more advanced plasmonic materials. The theoretical background of the SPR microscopy sensor measurements is provided in the recently published work of Zybin and colleagues [18].

Separate localization of individual particle signals enables particle concentration measurement and the sizing of particles. Once a region has been detected as a particle signal region, the changes of intensity value in the associated pixels over time indicate its relative size [6,19]. By measuring the relative size of particles with known optical properties, the conversion factor from relative sizes to absolute sizes is ascertained. If the optical properties are unknown, then a calibration with known particles (e.g., bio-similar particles, such as VLP or polymeric particles) is possible. The conversion of particle counts can be converted to an actual concentration, either by an absolute measurement [20] or a calibration with known particle suspensions. In this way, size and concentration can be simultaneously determined [6,19].

To date, evaluations have demonstrated the detection capabilities on the submicrometer scale, starting from 80 nm for biological or polymeric particles [5,21]. The limits of detection, in terms of size, depend on the contrast, i.e., the difference in the refractive index between solvent and particle [18]; therefore, NPs with a high refractive index—for instance, gold in an aqueous solution—can be visualized down to a size of 40 nm.

Commonly used for EV analysis, flow cytometry (FC) and nanoparticle tracking analysis (NTA) require biological labeling prior to the specific detection of EVs. In addition, conventional FC instruments often suffer from resolution and sensitivity problems [2]. SPR microscopy, as a real-time imaging technique without biological labeling, overcomes the constraints of FC and NTA [2]. Moreover, as previously reported [6], the functionalization of a gold sensor chip surface by cysteine-conjugated protein A/G permits the achievement of the oriented immobilization of an unmodified anti-target antibody, which enables the re-use of a sensor chip after eluting an analyte-capturing antibody layer: thus, captured EVs or other bioNPs can be pre-concentrated, sized, and quantified onto a gold chip surface during SPR microscopy measurements, and further characterized after elution from an SPR gold chip. However, a primary custom-made version of the SPR microscopy instrument still had inhomogeneous illumination of the gold sensor layer and a relatively small focusing area. These limitations hampered the ability of the SPR microscopy sensor to detect low concentrations of analyte bioNPs in solutions. Mounting a concave lens and a diffraction grating helped to correct the image distortion caused by tilted object and image planes in the primary version of the SPR microscopy sensor: thus, mounting these elements resulted in a more homogeneous illumination of the enlarged focused gold sensor surface area of the modified SPR microscopy instrument.

Among the aims of the current work was the necessity to provide detailed protocols describing the setup of the primary and modified versions of the SPR microscopy sensors, as well as principles of measurements for samples containing different types of NPs. It was important to confirm that employed modifications did not affect the main characteristics of the SPR microscopy sensors, such that, for the primary and modified versions of the SPR microscopy sensor, the character of dependency between NPs concentration and the number of binding signals detected by the sensor, as well as between the size of NPs and the intensity of binding signals, remained linear. We demonstrate the ability of a modified SPR microscopy sensor to establish a wide dynamic size range in detecting silica NPs (with a refractive index close to bioNPs) of sizes 200 nm and 800 nm on the same gold sensor chip simultaneously. We study the power of the modified and primary versions of SPR microscopy sensors in detecting EVs isolated via different approaches. We suggest the employment of actuators—precise stepper motors—that help to adjust the positions of the optical elements in the primary version of the SPR microscopy sensor. Finally, we discuss the principles of software development, which aim to establish efficient and reliable sensor–actuator coupling.

The structure of this manuscript is as follows: In Section 2, the setup of the SPR sensor, the preparation of the gold surface, and the samples are described; a description of the acquisition, analysis, and optimization of the prism rotation, or the camera lens shift based on it, is given; Section 3 shows the results of the previously described approaches and investigations. In Section 4, we discuss the presented results, and we present an outlook on future research perspectives.

## 2. Materials and Methods

In Section 2.1, we present the basic structure of the SPR sensor (Section 2.1.1), the mechanical components for the adjustment of the optical parts (Section 2.1.2), and the preparation of the gold sensor surface for SPR microscopy (Section 2.1.3). Section 2.2 gives details of culturing HT29 Cells (Section 2.2.1), the isolation of EVs produced by HT29 cells (Section 2.2.2), culturing human mesenchymal cells (Section 2.2.3), and the isolation of EVs from those cells (Section 2.2.4).

Section 2.3 describes the utilization of the SPR sensor for detecting wide ranges of nanoparticle sizes in one sample (Section 2.3.1), and the recording and analysis of nanoparticle images (Section 2.3.2). The creation of a comparative analysis by a commercial nanoparticle-tracking analysis device for determining particle sizes is discussed in Section 1.3 in Supplementary Materials.

In Section 2.4, we present our approach to optimizing the adjustment of prism rotation (Section 2.4.1) and camera objective translation (Section 2.4.2), to improve the conditions for downstream nanoparticle detection.

### 2.1. SPR Microscopy Sensor

#### 2.1.1. Assembling of the Flow Cell, SPR Sensor Prism, and Gold Sensor Plate for a Wide-Field SPR Microscopy Instrument

**Figure 1:**
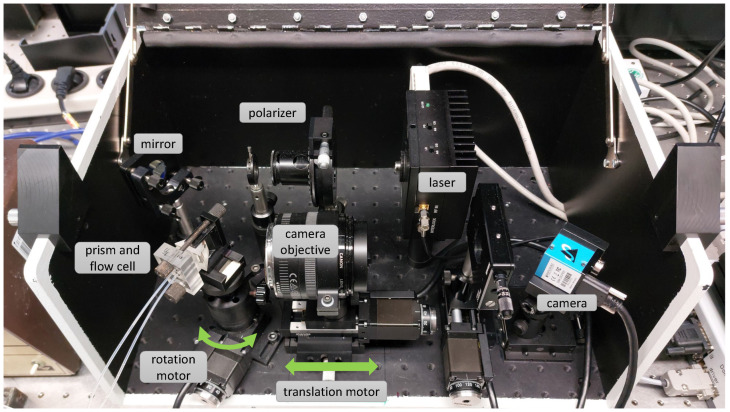
Schematic setup of the primary-version SPR microscopy sensor equipped with a rotation and a translation platform that can be controlled over two stepper motors connected via a USB controller.

We previously reported the ability of the primary-version SPR microscopy sensor to visualize the binding of inorganic nanoparticles (NPs) [11,12], as well as inactivated influenza A viruses or HIV-virus-like particles (HIV-VLPs) [7], to the functionalized sensor surface. This custom-made instrument, shown in Figure 1, employed Kretschmann’s scheme [3] of plasmon excitation.

**Figure 2:**
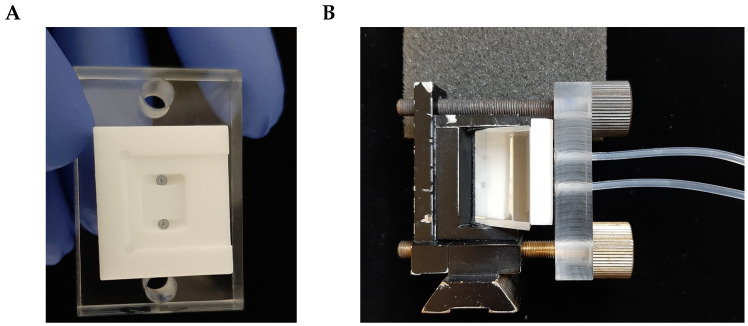
(**A**) a flow cell of the primary-version SPR microscopy sensor; (**B**) the assembly of a flow cell, a prism, and a prism holder ready to be placed at the rotation stage.

**Figure 3:**
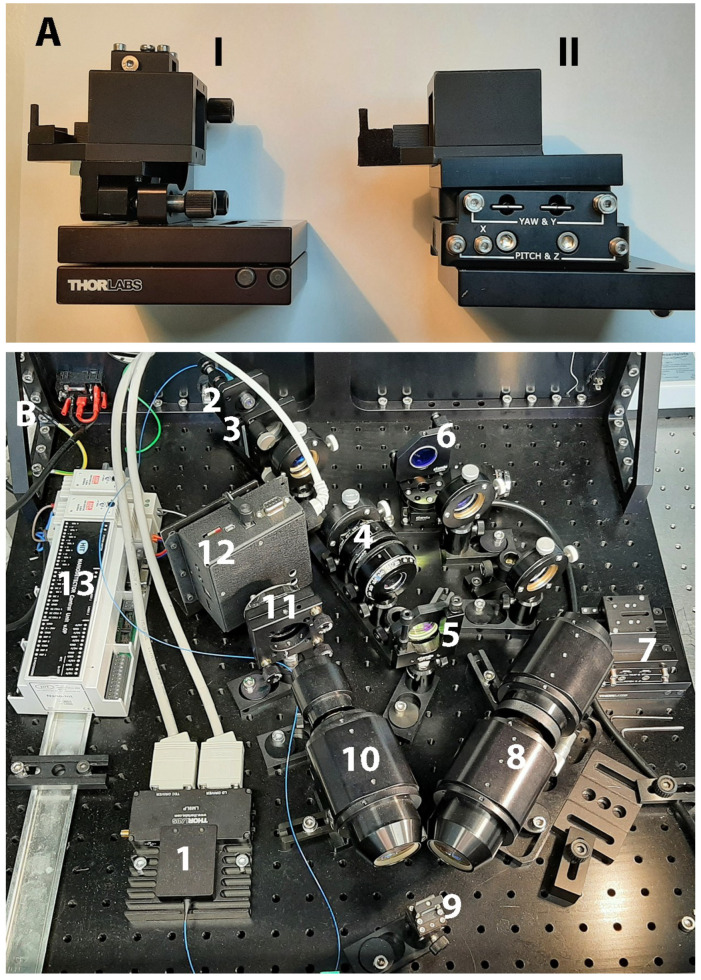
(**A**) (I) the prism and flow cell holder created for the modified version of the SPR microscopy sensor in the scope of the EC-FP7 project ”Nanodetector”, and (II) the improved version of such a holder, which includes a 5-axis platform; the 5-axis platform helps to fine-tune the prism and flow cell positions near the objective; (**B**) the internal view of the SPR microscopy sensor developed in the scope of the EC-FP7 project ”Nanodetector”. The following elements are presented: (1) an SM-fiber-coupled laser diode, (2) a diode head, (3) a concave lens, (4) a p-polarizer, (5) a mirror, (6) a scanning mirror mounted onto the motorized rotation stage, (7) a prism and flow cell holder, (8) and (10) focusing lenses (objectives), (9) a diffraction grating, (11) a CCD or CMOS image sensor chip, (12) a BeagleBoard-xM computer (www.beagleboard.org, accessed on 2 April 2023), (13) a controller.

However, the base of the SPR prism (SF10 glass, with refractive index n=1.72; 4 polished surfaces; 20×25×16.3 mm; 40-20scr/dig, SF l/2@ 633 nm, angle 56 deg ± 5’ produced by Eksma Optics, Vilnius, Lithuania, www.eksmaoptics.com, accessed on 2 April 2023) used for the studies mentioned above was not directly coated with a thin gold film, as described in Kretschmann’s original work [3]: instead, glass slides with the same refractive index and made from the same type of glass (SF10) were employed as a sensing surface carrying slides (the slides were produced either by Hellma Optics, Jena, Germany, or UQG (Optics) Ltd., Cambridge, UK). The glass slides (sizes 14×75×1 mm) were coated with a 5 nm adhesion layer of Ti and an approximately 41 nm-to-45 nm layer of gold. The deposition was performed using a magnetron sputtering technique (PHASIS, Geneva, Switzerland or Innolume, Dortmund, Germany). Cut glass slides—”gold sensors” (Section 1.3 in Supplementary Materials)—were placed on the prism base, using an immersion liquid with the same refractive index n=1.72 (Cargille Laboratories, Cedar Grove, NJ, USA). Then, an SPR prism with a gold sensor placed on its base was mounted into a flow cell (Figure 2A). The flow cell was custom-constructed and made from Teflon^®^. A polydimethylsiloxane (PDMS) gasket was used to prevent the flow cell from leaking (Section 1.1 in Supplementary Materials). Assembled this way, the flow cell, together with a gold sensor and prism (Figure 2B), was mounted on the small rotation stage (Thorlabs GmbH, Bergkirchen, Germany, Cat#KM100 or Eksma Optics Cat# 960-0170), which could be motorized, or on the static 5-axis platform (Thorlabs GmbH, Germany, Cat# PY005/M) (see Figure 3) in the case of the modified SPR microscopy sensor.

The initial steps of the flow cell assembling process are very similar for both the primary-version SPR microscopy instrument and its updated version (Figure 3), developed within the EC-FP7 project ”Nanodetector”. However, there are some differences: for example, for the improved version of the SPR microscopy sensor, smaller prisms are employed (SF10 glass prisms sizes 9×9×14 mm were produced by Eksma Optics as a part of the EU-Nanodetector equipment); moreover, as mentioned above, an SPR prism with a gold sensor has to be mounted on the static 5-axis platform, which helps to adjust the exact position of a prism and a flow cell near the objective. Thus, in the updated version of the SPR microscopy sensor, the prism, with an assembled flow cell and a gold sensor inside, remains motionless; in the primary version of the SPR microscopy sensor, the situation is the opposite: the prism is mounted on the rotation platform, and thus, can rotate.

#### 2.1.2. The Main Mechanical Parts of the Archetypal Hand-Constructed SPR Microscopy Sensor and Its Modified Version

**Figure 4:**
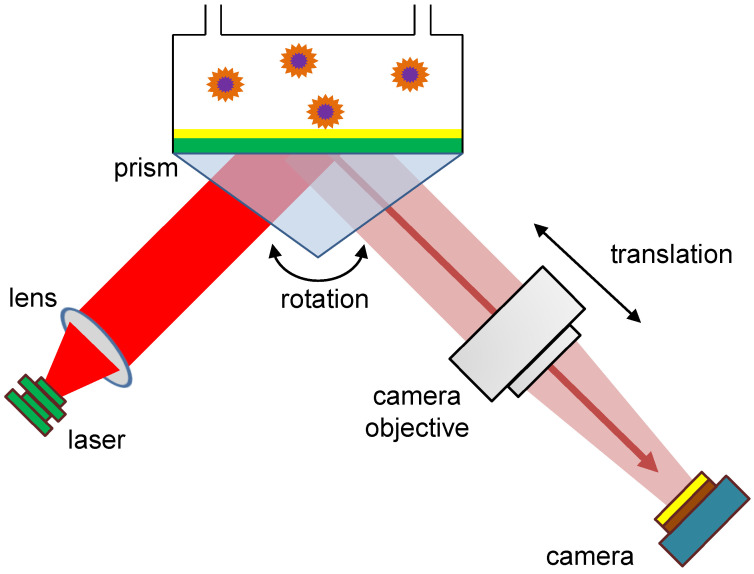
Schematic setup of the primary-version SPR microscopy sensor, including a rotation platform to change the incidence angle of the laser beamed at the prism and a translation platform to move the camera objective. Modified from [22].

In the Kretschmann scheme, as shown in Figure 4, the gold sensor layer can be illuminated through the glass prism by a collimated beam of the superluminescent diode or a laser diode. For the primary-version SPR microscopy sensor, a superluminescent diode (QSDM-680-9; QPhotonix, Ann Arbor, MI, USA) or a laser diode HL6750MG (Thorlabs GmbH, Germany) demonstrated their applicability [7]. Both diodes provided the excitation wavelength of the incidence beam λ=685 nm. In the case of the modified version of the SPR microscopy instrument, the wavelength used in the incidence beam was λ=642 nm, and an SM-fiber-coupled diode was employed (Thorlabs GmbH, Germany). For both instruments, current and temperature controllers from Thorlabs (Cat# LDC202C or LDC205C Benchtop LD current controller and Cat# TED 200C temperature controller) were employed. In the modified version of the SPR microscopy sensor, a beam was collimated by a 16 mm-focus-length objective (MVL16, Thorlabs GmbH, Germany) and was directed through a 14 mm-free-aperture Glan polarizer (from Eksma Optics, www.eksmaoptics.com, accessed on 2 April 2023), to create p-polarized light. In the setup of the primary-version SPR microscopy instrument, a Glan–Taylor polarizer (Cat# GT10-A; Thorlabs GmbH, Germany) was employed to achieve the p-polarization of the light illuminating the gold sensor surface. A 50 mm Minolta Rokkor MD photo-objective, with an aperture of 1/1.7, was used to image the gold surface signals with a video camera. An objective from Canon (Canon Compact Macro Lens EF 50 mm 1:2.5) could also be employed for the gold sensor surface imaging. Different CMOS and CCD image sensor chips have proved their power for detecting individual nanoparticles in SPR microscopy instruments. As with the primary version, the modified version of the SPR microscopy instrument used an MT9P031 CMOS image sensor chip with a resolution of 5 Megapixels; in the primary version of the SPR microscopy instrument, this CMOS chip was used in a DMK23UP031 camera, produced by Imaging Source (Bremen, Germany). This chip possesses a pixel size of 2.2×2.2 μm. However, for the primary version of the SPR microscopy sensor, a 5-megapixel GC2450 Prosilica camera (Allied Vision, Stadtroda, Germany), with a Sony ICX625 CCD image sensor and a pixel size of 3.45×3.45 μm, also proved its efficiency at detecting extracellular vesicles [6], viruses, and virus-like particles [7].

#### 2.1.3. Functionalization of the Gold Sensor Surface for Detecting Biological NPs Employing SPR Microscopy Instruments

**Figure 5:**
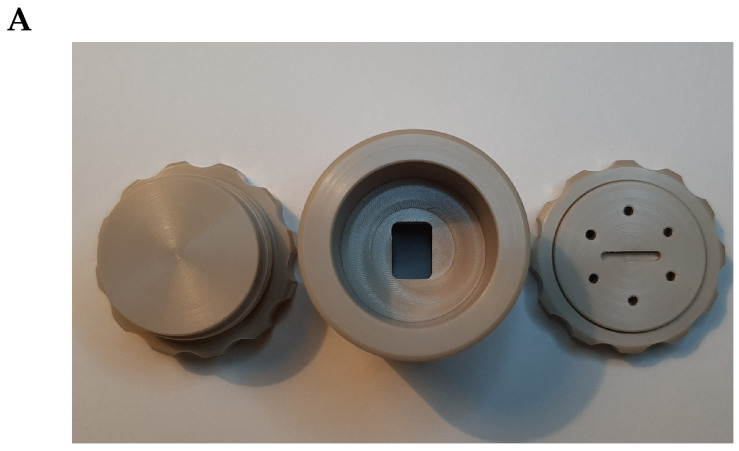

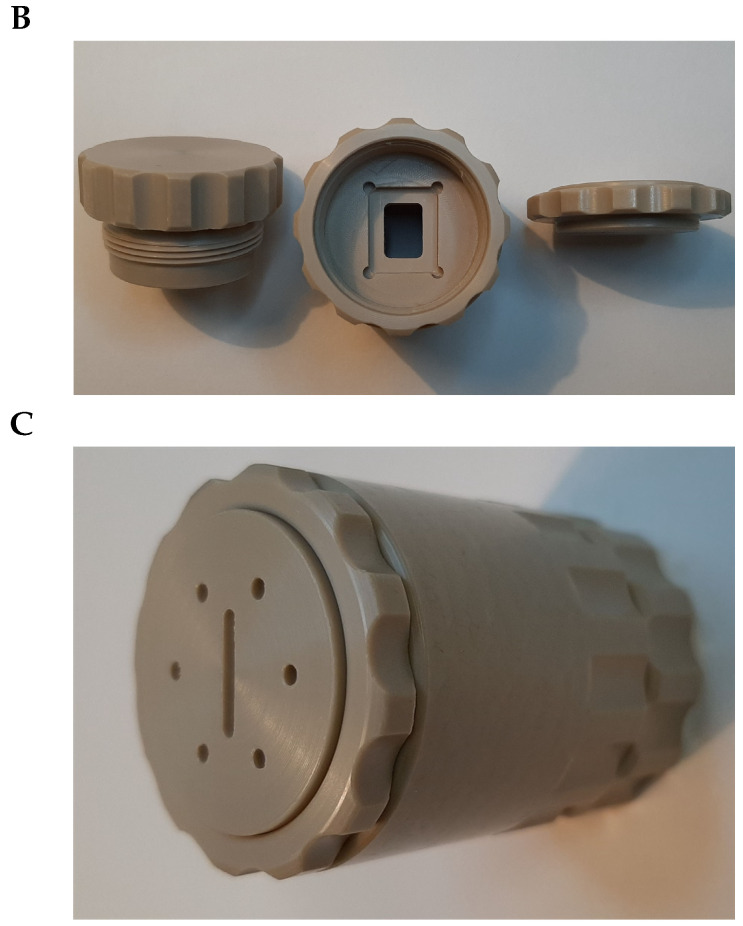
(**A**) the custom-made “coating chamber”: top view of a lid and a chamber; (**B**) bottom view of the “coating chamber” lid and the chamber itself, with a groove for a PDMS gasket and a gold sensor chip; (**C**) assembled “coating chamber”.

After cutting and cleaning the “gold sensors” (Section 1.1 in Supplementary Materials), sensor surface functionalization can be performed. The functionalization steps for the detection of inorganic nanoparticles (polystyrene and silica) are described in Section 1.2 in the Supplementary Materials. Here, we describe the steps for the detection of bioNPs. The following self-assembling monolayers (SAMs) are formed on the sensor surface: (1) the first layer is formed by cysteine-conjugated protein A/G (cys-protein A/G) (from BioTechne, Wiesbaden, Germany; Cat# NBP2-34862-5mg; www.novusbio.com, accessed on 2 April 2023); (2) the second layer is constituted by anti-target antibodies. For the detection of extracellular vesicles (EVs), anti-CD81 (Santa Cruz Biotechnology, Heidelberg, Germany, clone 5A6, Cat#sc-23962 or BD Biosciences, Heidelberg, Germany, clone JS-81, Cat#555675), anti-CD63 (BD Biosciences, clone H5C6, Cat#556019), and anti-CD9 (Dianova, Hamburg, Germany, clone VJ1/20, Cat#9PU-01MG or Santa Cruz Biotechnology, clone ALB6, Cat#sc-59140) antibodies can be employed. Moreover, using cys-protein A/G as an intermediator layer between the gold sensor surface and the layer of anti-target antibodies helps to re-use the same gold sensor at least three times [6]. The antibodies can be eluted with captured bioNPs, and replaced by the same or different antibodies [6]. Cys-protein A/G was used to coat a gold sensor surface at a concentration of 30 μg/mL. Anti-target antibodies (CD81, CD63, or CD9) were used in a concentration of 10 μg/mL to be immobilized on the cys-protein A/G layer for further capturing of EVs isolated from HT29 or human mesenchymal cells. Unbound cys-protein A/G and antibodies were removed via washing steps, which were performed by applying PBS (Pan Biotech GmbH, Aidenbach, Germany, w/o calcium and magnesium, pH = 7.4). The saturation stage of each monolayer formation was indicated by a uniform and temporally constant background image. Thus, the formation of each monolayer (cys-protein A/G and antibodies) could also be monitored on the SPR microscopy instrument. The coating of a gold sensor with cys-protein A/G and antibody was performed for approximately 40 min for each step. However, to minimize the time a gold sensor was placed in the SPR microscopy instrument, a custom-constructed ”coating chamber” was applied (see Figure 5). In this coating chamber, a gold sensor was located in such a way that only the gold surface, not the glass one, was exposed to the PBS solution containing cys-protein A/G or antibodies. The coating could be performed at room temperature (RT) for 2 h on a shaker (CAT Ingenierbüro M. Zipper GmbH, Germany, Model#S20) at approximately a speed of 300 movements per minute or overnight at +4 ${}^{\circ }C$. Exposure of the glass surface to the protein-containing solution would have resulted in problems during the SPR microscopy measurements.

### 2.2. Cell Culture

#### 2.2.1. Culture of HT29 Cells and Collection of the Cell Culture Medium Containing Extracellular Vesicles (EVs)

HT29 cells were thawed at passage 6, seeded in a monolayer at a density of 5000 cells per cm${}^{2}$, and further expanded in McCoy’s Medium A5 supplemented with 1% penicillin/streptomycin and 10% heat-inactivated FBS (Sigma-Aldrich, Darmstadt, Germany). The cells were kept under standard culture conditions (37 ${}^{\circ }C$ and 5% CO${}_{2}$) in a humidified incubator, and medium exchange was performed every 2–3 days. The HT29 cells were maintained in a complete culture media. When the HT29 cells reached 80% confluence, the old culture media was aspirated, and the cells were washed with pre-warmed (37 ${}^{\circ }C$) sterile PBS twice, and once with complete culture media containing 10% heat-inactivated and exosomes-depleted FBS (Gibco via ThermoFisher, Cat#A2720803): this process eliminated the presence of EVs of bovine origin. Afterward, the cells were cultured with the same media for 48 h before the collection of conditioned media (CM). The CM aliquots were centrifuged at 3000× *g* for 10 min, and the supernatants were transferred to new tubes without disturbing the pellet: this step was essential to excluding cell debris and apoptotic bodies. The supernatants received this way were divided into three portions, each containing 20 mL. The portions were filtered using 1.2,0.45,0.22 μm filters to collect EVs of different sizes. CM samples were kept at −80 ${}^{\circ }C$ for further processing in EV isolation, using different approaches.

#### 2.2.2. Isolation of EVs Produced by HT29 Cells

To evaluate EV isolation based on membrane affinity spin columns, we employed an exoEasy Maxi kit from Qiagen and followed the isolation steps recommended by the manufacturer. In brief, frozen CMs were thawed and then re-filtered to remove cryo-precipitate. CM was mixed in a 1:1 ratio with XBP buffer, with further gentle inverting at room temperature. The XBP/CM mixture was added to the exoEasy spin column, centrifuged at 500× *g* for 1 min, and then the flow-through was discarded: this step was repeated until the whole volume was centrifuged. Next, 10 mL of washing buffer was added to the membrane, centrifuged at 5000× *g* for 5 min, and the flow-through was discarded. The spin column was then transferred to a new collection tube. A 400 μL volume of Elution buffer was added to the membrane of the spin column, and the column was centrifuged at 500× *g* for 5 min. Another 400 μL of elution buffer was added to the membrane and was centrifuged at 5000× *g* for 5 min. Aliquots of 100 μL of EVs in elution buffer were kept at −20 ${}^{\circ }C$ for further processing in SPR microscopy measurements. Another approach, which was also tested, is based on the ability of certain compounds to tie up and force less-soluble EVs out of the solution. We applied, for this type of EV isolation, Total Exosome Isolation (from cell culture media) reagent (Invitrogen): this reagent helps to concentrate EVs from cell culture media after overnight incubation with collected media samples, followed by their centrifugation at 10,000× *g* for 1 h at 2 ${}^{\circ }C$ to 8 ${}^{\circ }C$.

#### 2.2.3. Culture of Human Mesenchymal Cells (MSCs)

Human bone marrow (BM) aspirates from healthy donors were obtained, following informed consent according to the Declaration of Helsinki. Their usage was approved by the ethics committee of the University of Duisburg–Essen (12-5295-BO). To raise MSCs, aliquots of the obtained BM aspirates were seeded into cell culture flasks containing endothelial basal media (EBM-2, Lonza, Köln, Germany), supplemented by 10% human platelet lysate (PL; produced by the working team of Prof. Dr. Giebel), and provided by a Lonza bullet kit. After incubation for 24 h at 37 ${}^{\circ }C$ in a 5% $CO_{2}$ atmosphere, non-adherent cells were removed by the medium exchange to DMEM low glucose (PAN Biotech GmbH, Aidenbach, Germany), supplemented by 10% PL, 100 U/mL penicillin–streptomycin–glutamine (Thermo Fisher Scientific, Darmstadt, Germany), and 5 IU/mL heparin (RatioPharm, Ulm, Germany). The cells were continuously cultured at 37 ${}^{\circ }C$ in a 5% $CO_{2}$ atmosphere and were regularly screened microscopically until the first MSC colonies became visible. Following trypsin/EDTA (Sigma-Aldrich, Darmstadt, Germany) treatment, including a washing step, the adherent cells were re-seeded, at densities of approximately 1000 cells per cm${}^{2}$, into 4-layer-stack cell factoryTM systems (Thermo Fischer Scientific GmbH, Schwerte, Germany). Within the second passage, the MSCs were analyzed according to the criteria of the International Society of Cell and Gene Therapy (ISCT) [23]. Upon reaching densities of approximately 50% confluence, the conditioned media (CM) were changed every 48 h. At 80% confluence, the MSCs were passaged. Before preserving the CM, cells and larger debris were removed via centrifugation at 2000× *g* for 15 min. MSC-free CMs were stored at −20 ${}^{\circ }C$.

#### 2.2.4. Isolation of EVs from MSCs

For EV harvesting, the CM were thawed and further purified, following 45 min 6800× *g* centrifugation (Rotor: JS-5.3; Beckman Coulter), by a subsequent 0.22 μm filtration step using rapid-flow filter (Nalgene, Thermo Fisher Scientific). The EVs were precipitated in 10% polyethylene glycol 6000 (PEG) and 75 mM sodium chloride (NaCl) by overnight incubation and subsequent centrifugation at 1500× *g* and 4 ${}^{\circ }C$ for 30 min, as described previously [24,25]. The pelleted EVs were re-suspended and washed with a sterile 0.9% NaCl solution (Braun, Melsungen, Germany). Next, the EVs were re-precipitated by ultracentrifugation at 110,000× *g* for 130 min (XPN-80, Ti45 rotor, k-factor: 133). Finally, the EV pellets were re-suspended in 10 mM HEPES 0.9% NaCl buffer (Thermo Fisher Scientific, Darmstadt, Germany). MSC–EVs preparations were stored at −80 ${}^{\circ }C$ until use in SPR microscopy experiments.

### 2.3. Image Processing and Analysis

#### 2.3.1. Detecting the Binding of Polystyrene or Silica Particles to the Gold Sensor Surface of the Modified SPR Microscopy Sensor

For the detection of the binding of polystyrene 100 nm, 200 nm, 300 nm, and 400 nm in diameter, or silica nanoparticles 200 nm and 800 nm in diameter, to the gold sensor surface, gold sensors functionalized with “Nüscoflock” were used (see Section 1.1 in Supplementary Materials). Prior to the measurements, all NP suspensions were incubated in an ultrasonic water bath Elmasonic S10-H (Elma, Singen, Germany) for a duration of 10–15 min to eliminate particle agglomerates. Nanoparticles were pumped through the flow cell as a suspension in distilled water containing 0.1% sodium chloride. For pumping, silicon tubing (IDEX Health and Science, Germany; ID of the product: 0.48 mm; color orange/yellow) and a peristaltic pump Rabbit, Peristaltic Pump (4-channel; Rainin Instruments, France) were used. Pumping was performed at a speed of 0.3 mL/min. Before and after each experiment, the flow cell system was washed with PBS (for the measurements of extracellular vesicles) or distilled water containing the appropriate concentration of sodium chloride (for the measurements of silica or polystyrene NPs). For the visualization of NP binding events, the beam incidence angle was chosen on the left (smaller angle) slope of the reflected intensity curve, near the minimum of the reflected intensity (resonance angle) [18]. Therefore, the highest signal-to-noise ratio was expected at an angle close to the minimum of the reflected light intensity [18]. Thus, measurements of inorganic NPs and bioNPs were performed at approximately 0.1 degrees before the SPR minimum. However, a different position was chosen for the visual control of SAM formation on the gold sensor surface: these measurements were performed at approximately 0.4 degrees before the SPR minimum.

#### 2.3.2. Nanoparticle Image Recording and Image Processing

**Figure 6:**
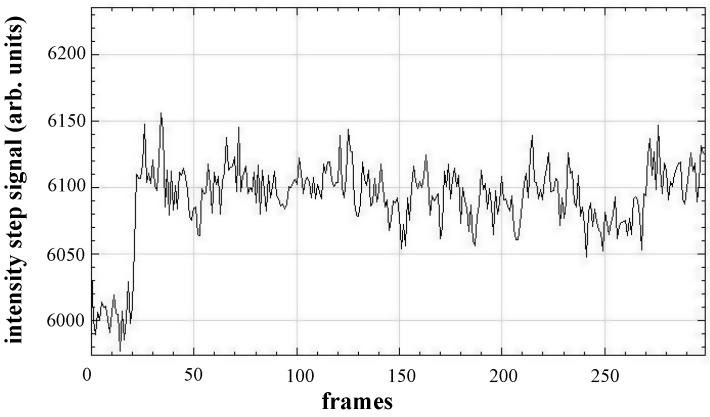
A typical profile of a local image intensity change caused by the binding of a 200 nm silica nanoparticle bound to the gold sensor surface of the modified SPR microscopy instrument. Adapted from [26].

A characteristic feature of a nanoparticle binding event in the processed images is intensity changes—“step” signal—(see Figure 6), which help to confirm the particle binding. This feature can be easily noticed during manual image processing (see Section 1.2 Supplementary Materials).

**Figure 7:**
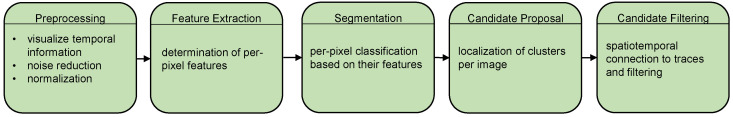
Meta-pipeline for the detection of nanoparticles in image series. Several approaches share similar steps in their specific pipelines [22].

However, manual processing of the recorded images is an ineffective procedure. Thus, different methods for analyzing recorded images have been developed and described [22]. Recent improvements are based on machine learning methods and exploit their adaptivity to learning a tailored analysis from training data in order to find the characteristic features of particle regions [27,28,29,30]. Although individual approaches differ, they can usually be assigned to a meta-pipeline, as illustrated in Figure 7. The first step is preprocessing, which renders particle signals expressed in time by the changed reflection behavior visible spatially. Next, characteristic features are extracted for each pixel or region of the image: This information is used to segment pixels whose features suggest particles of interest based on the features of their vicinity. Segmented clusters that match given criteria are then detected as candidates for single representatives of particles of interest. As those particles are not only visible on one image but over multiple frames, the candidates per image are connected spatiotemporally, so that multiple local detections form a particle trace. As the final step, non-plausible traces are filtered out based on spatial, temporal, or spatiotemporal criteria, and the remaining traces are seen as actual particles of interest.

An example of a concrete implementation is an approach that uses a U-Net [31] on preprocessed images to generate features and segment them in one module [27]. The candidate proposal is achieved via a Difference-of-Gaussian-based detector [32], which processes the output of the U-Net. Next, the candidates of all the images are connected over time and filtered based on their length and missing frames in between multiple detections at one image region. As optical disturbances appear in each recording and can also be seen in different types—e.g., random noise, fixed-pattern noise, wave-like, or line-like artifacts [27]—we developed an approach for achieving high robustness against image artifacts [27]. In this approach, a U-Net was trained on real examples showing particles of interest combined with disturbance patterns. These patterns were generated by a generative adversarial network (GAN) [33] that was trained on real disturbance patterns and was used to create an arbitrary amount of synthetic but realistic-looking images containing artifacts. By this method, the robustness of the detection against imaging artifacts increased [27]. In the following, we also use this analysis method as a metric for optimizing the settings of the motors for reliable detection of particles.

### 2.4. Optimization of Adjustments via Sensor–Actuator Coupling

When preparing the SPR microscopy sensor for analysis, it must be adjusted beforehand: this means that after preparing the flow cell, the position of the camera objective and the rotation of the prism must be adapted to the current experimental requirements. In a typical experiment, the prism rotation and the camera objective translation are done by manual control of two stepper motors, which are shown schematically in Figure 4, and as an actual setup in Figure 1. The stepper motors (Eksma Optics, Lithuania, www.eksmaoptics.com, accessed on 2 April 2023) employed for the adjustments in the primary version of the SPR microscopy instrument are the following: (1) motorized rotation stage small precision of 0.9 arcmin (Art.# 960-0170); (2) narrow motorized translation stage (Art.#960-0060). In order to control these motorized stages, a one-axis USB controller (Art.# 980-1045) was used. Aiming to reduce the need for manual interaction, we describe an approach to measuring the quality of the recorded images, tailored to the given task of nanoparticle detection: We propose an automation prototype based on that measure and evaluate it on sample recordings, with different configurations of the motorized sensor platforms.

#### 2.4.1. Adjusting the Prism Rotation

One factor in an optimal configuration to make nanoparticles detectable is the rotation angle of the prism, which influences the contrast between the particles of interest and background signals. At this point, a solution containing 0.1% sodium chloride in distilled water, but without particles of interest, is used for calibration. By rotating the prism, we approximate the angle of total reflection [34] under which the reflected laser beam has the lowest intensity while still hitting the camera chip. As the analyzed solution does not contain any particles of interest, the intensity of the background signals is minimized. The possible range of rotation is given relatively, including only the positions in which two conditions are fulfilled: the laser hits the camera chip, and there is no risk that the prism rotation will physically pull the tubing for pumping the fluid through the flow cell.

#### 2.4.2. Adjusting the Camera Objective Translation

**Figure 8:**
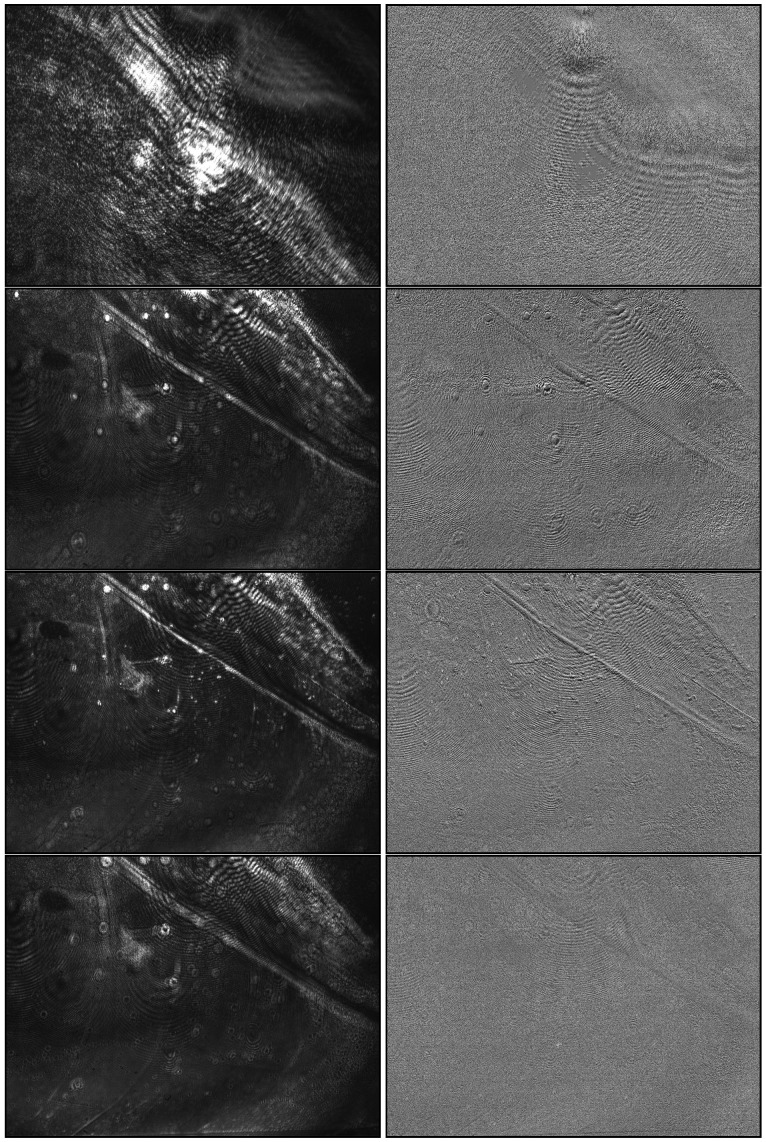
Images recorded at four relative objective positions (0.00, 0.64, 0.68, and 0.72, top to bottom) for a sample containing 300 nm polystyrene particles. The left side shows raw images and the right side shows preprocessed images generated in the same positions. The images of position 0.68 contain the focus area, where particle signals can be seen as bright dots.

The second optimization determines the focus area by the position of the camera objective, and is characterized by particles being imaged as sharply as possible: this means that low distortion of the spots showing particle signals in preprocessed images provides optimal conditions for downstream particle detection. As particle detection is the main task and the reason for improving the adjustments, the detection method is used to measure image quality. Preprocessing is needed to make particles of interest visible for image analysis methods by extracting the changes in the signals over time instead of using the absolute intensities on each image. Figure 8 shows raw and preprocessed images for selected example positions, containing in-focus and out-of-focus examples. As this approach relies on particle signals, sample particles are needed for calibration, and each position has to be held to record a sufficient amount of images. The reason for this is that particles attach over time: A longer acquisition time per position improves the accuracy but slows the overall process; a higher concentration of particles in the calibration sample can, on the other hand, reduce this time. In each case, the time requirement is relativized by the fact that the calibration process does not have to be repeated for each follow-up analysis.

**Figure 9:**
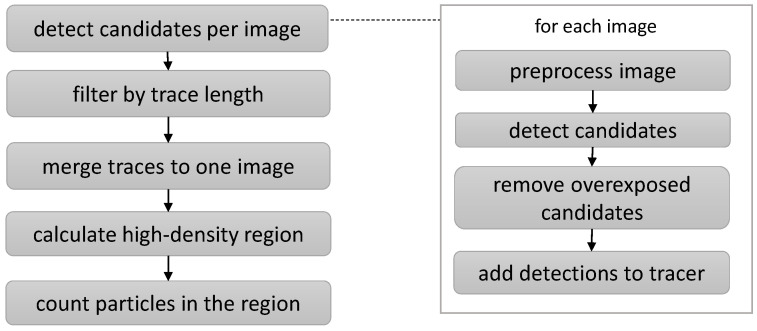
Visualization of the focus region detection for one motor position. By analyzing multiple frames and linking the detections of individual images over time to form traces, improved stability can be achieved.

Focus determination is executed in the steps visualized in Figure 9. The steps of focus detection are performed in each of the chosen positions of the motorized platform so that one focus region candidate, and the number of particles detected in it, are determined for each position separately: the latter is relativized to the count per 100 images, and is used as a metric in the overall detection of the optimal positioning; the following paragraphs detail the individual steps.

##### Preprocessing

In order to make particles visible for detection and evaluation of the focus, the acquired raw images are first preprocessed to emphasize signal changes instead of absolute intensities. This step is undertaken separately for each position of the translation stage. Recording *n* raw images $R_{0},\ldots ,R_{n-1}$ with $R_{t}\in \left[0,1\right]$ for t∈[0,n−1] in a fixed sample position, the first raw image $R_{0}$ serves as a reference to visualize the changes

(1) $$I_{t}:=R_{t}/R_{0}$$

and, by that, also the particle adhesions from a subsequent raw image $R_{t}$ for t>0. In detail, increases in pixel intensities, such as those caused by particles of interest, are transformed to high values, while other signals should show lower values. For better visibility, static contrast enhancement [28], with a factor of α=3 and a clipping of extreme values, is applied before further use.

##### Detection of Particles

In order to detect particle candidate regions in a preprocessed image, we used a previously developed approach based on a 5-layer U-Net [31] architecture, with 8 filters in the first layer. The way it was trained, as described in Section 2.3.2, optimized it to be robust for imaging artifacts: in this way, false detections are significantly reduced [27] so that the detection of the focus region becomes more reliable.

##### Removal of Overexposed Candidates

**Figure 10:**
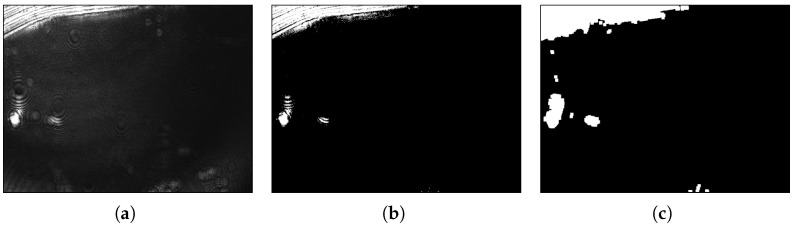
(**a**) example of a raw image, (**b**) the map of overexposed pixels created from the raw image by thresholding, and (**c**) a map with dilated overexposed regions for filtering out false detections at the borders of gold foil defects. The thresholding classifies each pixel of the raw image with over 50% of the possible maximum as an overexposed region. The maximum filter for dilation has side lengths of 32.

No image taken by the sensor can be assumed to be free of disturbances [27]. One type of disturbance that can be reduced directly, based on domain knowledge, originates from film defects, which are the main reason for local flickering effects visible in the raw image as overexposed, i.e., bright regions. In the preprocessed images, the flickering is visible in the direct vicinity of defect regions and can lead to false detections. The overexposed pixels are determined based on a previous method by Libuschewski [35]: first, a binary map is created, using a brightness threshold that marks overexposed pixels as 1 and others as 0; then, the white areas are dilated with a maximum filter of fixed side lengths $d_{dil}$, to include the neighborhood of defects; next, those candidate boxes from the previous step that overlap the dilated map of overexposed regions are filtered out. An example of this process is shown in Figure 10.

##### Tracing

Particles of interest can be seen on multiple frames of the preprocessed images so that a detection *b* in one image is only considered a candidate of such a particle. As the attached particles hardly move spatially over the acquisition period, the candidates detected on previous images are linked over time [28]. Two detected regions, $b_{1}$ and $b_{2}$, that appear in consecutive images are therefore linked to form a trace if they have a Jaccard index

(2) $$J\left(b_{1},b_{2}\right)=\frac{|b_{1}\cap b_{2}|}{|b_{1}\cup b_{2}|}$$

of greater than $j_{\min}$. A trace $s_{i}=<b_{i_{0}},b_{i_{1}},\ldots ,b_{i_{n-1}}>$ is extended by another detection $b_{k}$ if $J\left(b_{n-1},b_{k}\right)\geq j_{\min}$. This process continues across all detections until no more merges are possible. To account for short-term missing detections that would otherwise cause a link to be missing, we use the function $f^{\left(\mathrm{idx}\right)}$ to represent the index of the frame that contains a detection *b*. A temporal gap of $d_{\mathrm{tol}}$ frames between two detections is tolerated to connect them to one trace. This means that $\forall k<n-1:f^{\left(\mathrm{idx}\right)}\left(b_{i_{k}+1}\right)-f^{\left(\mathrm{idx}\right)}\left(b_{i_{k}}\right)\leq d_{\mathrm{tol}}$ holds for each accepted trace $s_{i}$ of length *n*. When all traces are constructed, we remove each trace that is visible on too few frames. In detail, an accepted trace $s_{i}$ of length *n* has to fulfill $f^{\left(\mathrm{idx}\right)}\left(b_{i_{n-1}}\right)-f^{\left(\mathrm{idx}\right)}\left(b_{i_{0}}\right)+1\geq n_{\min}$.

##### Determination of the Focus Region and Focus Metric

The focus for one motor position can be calculated with the traces $S=\{s_{0},s_{1},\ldots ,s_{m-1}\}$ that remain after filtering. For each trace $s=<b_{0},b_{1},\ldots ,b_{n-1}>$, the pixels included in any $b_{j},0\leq j<n$ are used to create an image $I^{\left(\mathrm{merge}\right)}$ that combines all traces in one image. In this image, each pixel *p* is marked as

$$I^{\left(\mathrm{merge}\right)}\left(p\right)=\{\begin{array}{cc} 1, & \mathrm{if}\;\exists s\in S:\exists b_{i}\in s:p\in b_{i}\\ 0, & \mathrm{otherwise}. \end{array}$$

**Figure 11:**
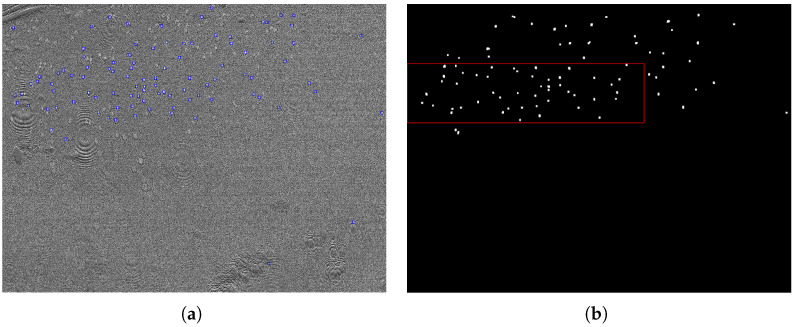
(**a**) example of a preprocessed image showing single-image particle detections for a sample containing 300 nm polystyrene particles as blue boxes, and (**b**) the corresponding merged map $I^{\left(\mathrm{merge}\right)}$ showing the merged traces calculated on the whole set of preprocessed images. The determined focus region is marked by a red box.

Then, a set of overlapping boxes of size $\left(x^{\left(\mathrm{select}\right)},y^{\left(\mathrm{select}\right)}\right)$ is extracted from $I^{\left(\mathrm{merge}\right)}$, and the mean value of all the pixels is calculated for each region. The region with the highest value is considered a candidate focus region, while the number of detected particles in it is used as a measure for the respective motor position. An example of this step is illustrated in Figure 11.

##### Overall Process

The method described above is applied to each motor position separately. In each position, preprocessing, detection, and removal of overexposed regions are performed for each image recorded in that motor position. With all detections combined to traces, the focus region and its measure are calculated per motor position, based on the image $I^{\left(\mathrm{merge}\right)}$ of merged traces. When the focus information is available for each position, the highest value is chosen to set the optimal motor position, focusing on the particles of interest. The determined box in the optimal motor position is considered the focus area.

## 3. Representative Results

### 3.1. Ability of the Modified SPR Microscopy Sensor to Detect Individual EVs

**Figure 12:**
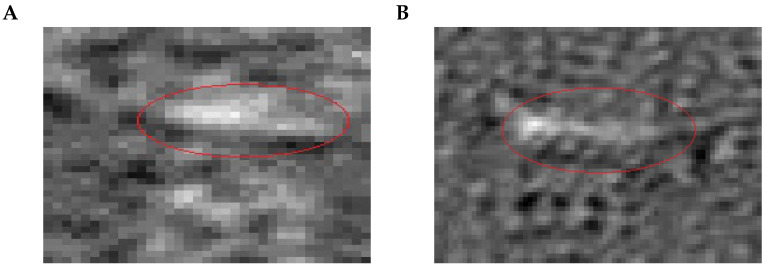
(**A**) for this sample, the isolation of EVs from HT29 cells employing affinity spin columns was performed; before EVs isolation, cell culture supernatants were filtrated via 0.45 μm filter (see Methods for the details); (**B**) for this sample, the EVs were prepared by adding to the collected cell culture supernatants a special buffer, which helped to tie up and to force the EVs out of the solution (details provided in Methods). The thin-line red ellipses mark vesicle binding signals. The long axis of the red ellipse on panel A has a size of approx. 8.36 μm. The long axis of the red ellipse on panel B has a size of approx. 15.96 μm.

Previously, it was reported that a primary version SPR microscopy sensor is capable of visualization of the binding of individual microvesicles (a subgroup of EVs) [6] harvested from cell culture supernatants after direct ultracentrifugation (100,000× *g* and 4 ${}^{\circ }C$ for 2 h 10 min). However, it remained unclear whether EVs can also be detected by a modified SPR microscopy sensor and whether EVs isolated via other approaches can be detected. In the present work, we studied the ability of a modified SPR microscopy instrument to detect individual EVs isolated through affinity chromatography and sedimentation via the exclusion of water molecules or PEG + UC. For the formation of SAMs on a gold sensor chip surface, we employed the “coating chamber” or formed SAMs under flow conditions in the flow cell of the SPR microscopy sensor. The results of measurements performed with EVs isolated by different approaches are presented in Figure 12. It is important to note that both the affinity chromatography approach and sedimentation via water exclusion generated EV samples, in which signals from individual EVs were detectable by the SPR microscopy sensor. However, the PEG + UC approach did not lead to the isolation of vesicles detectable by the SPR microscopy sensor (data not shown).

### 3.2. Wide Dynamic Particle Size Range of the Modified SPR Microscopy Sensor

**Figure 13:**
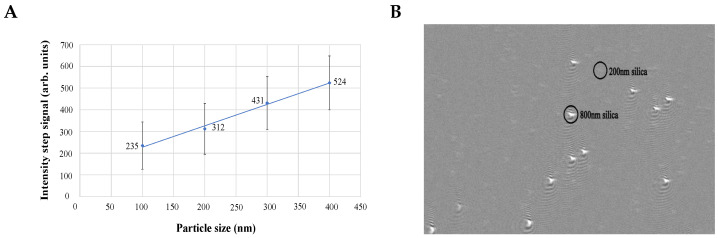
(**A**) dependency of the value of the intensity changes (“step” signal) from the size of nanoparticles; for these measurements, polystyrene particles of sizes 100 nm, 200 nm, 300 nm, and 400 nm were used; three independent experiments were performed; (**B**) 800 nm and 200 nm silica particles were detected simultaneously by the modified SPR microscopy sensor; the black circles mark the particle binding signals. Adapted from [26].

The necessity to compare the characteristics of the modified SPR microscopy sensor to those of the primary custom-made version of the instrument was among the aims of the current research work. Previously, the linearity of dependency between the concentration of NPs in suspension and the number of particle binding signals was demonstrated for the primary-version SPR microscopy sensor [12]. Moreover, the linear dependency between the size of measured NPs and the intensity of received SPR signals was also reported [11]. Together, these results indicated that the primary version SPR microscopy sensor, after calibration, could be employed for the sizing and quantification of NPs in solutions. In the current work, we confirm that the modified SPR microscopy instrument also shows the linear dependency between the size of analyzed NPs and the intensity value of a received "step" signal (Figure 13). The results of our experiments with different NP concentrations also confirmed linear dependency between the concentration of NPs in the analyzed sample and the number of signals detected by the modified SPR microscopy sensor (data not shown).

Furthermore, we demonstrated that the modified SPR microscopy sensor has a wide dynamic particle size range and enables the simultaneous detection of NPs of 200 nm and 800 nm. In these experiments, silica nanoparticles of 200 nm and 800 nm in diameter were used. The refractive index (RI) of the employed silica nanoparticles is rather close to that of biological nano-vesicles: $RI_{\mathrm{silica}}$ is around 1.47 [36]; $RI_{\mathrm{bioNPs}}$ is 1.37–1.42 [37]. Thus, silica nanoparticles resemble the optical characteristics of bioNPs and may serve as their model in SPR microscopy studies. On the other hand, particles in such a size range may closely mimic the size ranges of different neighboring biological objects, such as bacteria and microvesicles, which may be produced either by bacteria or by eukaryotic cells in response to contact with bacteria. Thus, simultaneous detection of silica NPs of such sizes indicates the potential applicability of the SPR microscopy instrument to visualization of the synchronous binding of microvesicles and bacteria to the gold sensor surface. Indeed, the results of our experiments performed on the modified SPR microscopy instrument (Figure 13) demonstrated the possibility of visualizing the binding of 200 nm and 800 nm silica particles to the gold sensor surface.

### 3.3. Changes in the Magnification Factor for the Modified SPR Microscopy Sensor

**Figure 14:**
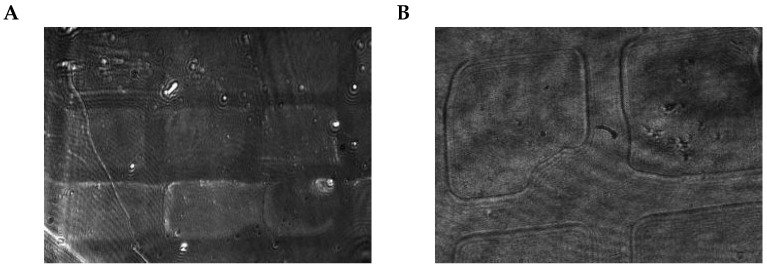
(**A**) the image of the nickel grid placed on a gold sensor surface of the primary-version SPR microscopy instrument, and (**B**) the image of the same grid placed on the gold sensor surface of the modified SPR microscopy instrument. Adapted from [26]. The nickel wire thickness, visible as the distance between the squares, was calibrated to 40 μm.

In both the primary-version and modernized SPR microscopy instrument, an objective with a low numerical aperture (NA) is used: this approach helps to increase the field of view on the gold sensor chip surface, where NP binding signals can be detected and means that the instrument is capable of analyzing lower NP concentrations in samples. However, the Scheimpflug principle affects the composition of elements (prism, objective, and camera) in such an optical design. In the primary version of the SPR microscopy instrument design, the Scheimpflug intersection is located at a finite distance, but in the modified SPR microscopy sensor, the Scheimpflug intersection is compensated. The latter issue helps to avoid the necessity of a noticeable tilt between the camera and the objective, thus simplifying the positioning of the elements in relation to one other [5]. In turn, the implantation of a concave lens in the optical design of the modified SPR microscopy instrument enlarges the laser beam’s spot and consequently helps to illuminate a larger gold sensor surface. The intercalation of a collimator adjusts the beam parallel. Together, these elements help to illuminate the gold sensor surface more homogeneously. Following these modifications, it was important to define the magnification factor of the modified SPR microscopy sensor. For this purpose, a nickel grid (dimensions 10 mm long, 4 mm wide, and 40 μm wire width) was inserted over the gold sensor surface. Images of this nickel grid, received for the primary-version SPR microscopy sensor and the modified version, are presented in Figure 14. The pixel size of the camera used in both cases for image recording is known: 2.2 μm× 2.2 μm. The magnification factor of the optical system was calculated as the ratio between the pixel size of the camera and the pixel size of the recorded image expressed in micrometers. Taking into account the parameters of the employed grid, one can calculate pixel dimensions for both sensors. The calculated image pixel width and height for the primary version sensor are 0.69 μm and 0.95 μm, respectively. The calculated image pixel width and height of the modified SPR microscopy sensor are 0.39 μm and 0.37 μm, respectively. Thus, the magnification factor of the modified SPR microscopy instrument employing an MT9P031 CMOS image sensor chip is approximately 4.5 times higher than that of the primary version sensor.

### 3.4. Results of Adjusting the Rotation of the Prism

**Figure 15:**
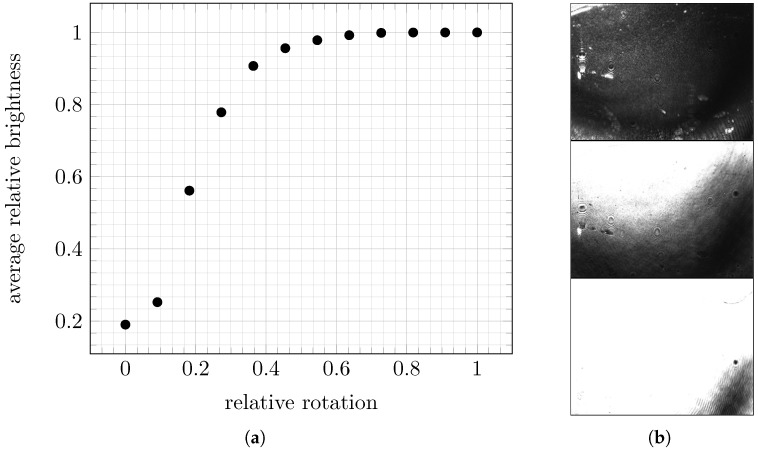
Plot (**a**) shows the average intensity of the recorded images while rotating the prism to different angles, and three examples (**b**) of raw images recorded at positions 0.0 (top), 0.18 (middle), and 0.45 (bottom). The angles are given relative to the maximum of the possible interval.

Figure 15 visualizes the results of an experiment where different rotations of the prism are set step by step.

A manual determination of the optimal angle was performed by an expert in addition to the automatic measurement. In this context, we use the term ”expert” to refer to laboratory personnel who regularly calibrate the instrument manually. An optimum close to 0 was determined at a manual operation, corresponding to the automatically found minimum. The optimal position can shift after a setup change, but the presented method can find the new optimum automatically or support manual operation.

#### Results of Automatic Focus Determination

**Figure 16:**
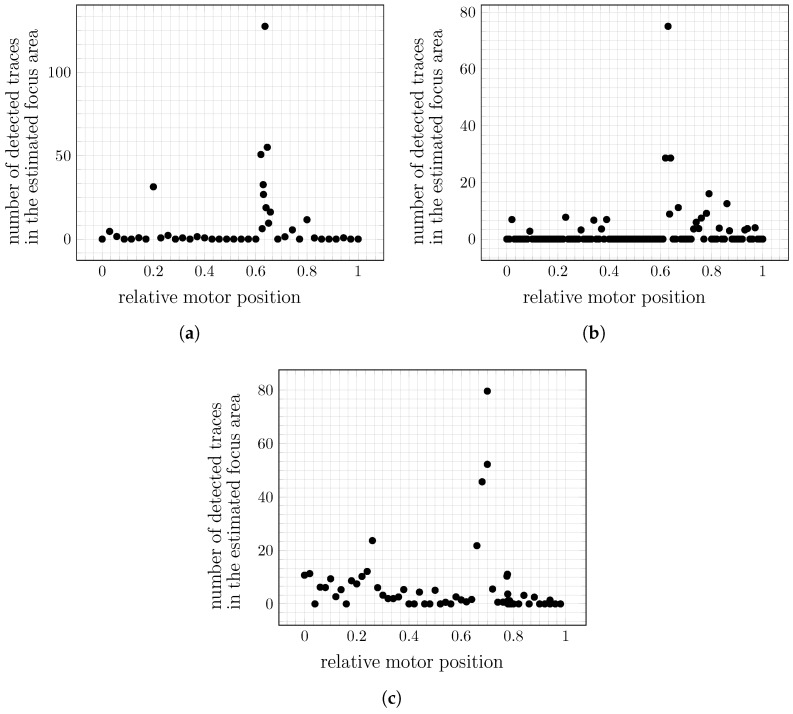
Sum of particle traces detected in the proposed focus region of each motor position. The detection follows the process described in Figure 9. The positions showing focus regions were determined by an expert at approximately (**a**) 0.64, (**b**) 0.63, and (**c**) 0.68.

We evaluated the presented approach for automatic focus determination in three experiments in which at least 25 images were recorded at each of the sample positions. Figure 16 shows the determined focus values originating from the presented method, using the parameter assignments of Table 1.

Taking the position with the highest value of the detection metric as the position of choice for each experiment, we find that they are consistent with the observations of an expert. This shows that the method can significantly reduce manual effort during the calibration of the optics. More specifically, the user can request the automatic calibration and can subsequently check whether the optimum has been adjusted correctly. Even in the case of a fine readjustment, the analysis results can be used as a suggestion for the range to search in, reducing the time needed for the manual operation that would otherwise have to be performed without a previous suggestion.

## 4. Discussion and Outlook

There is a clear need for new methods of analyzing biological NPs, such as viruses or extracellular vesicles. PCR (polymerase chain reaction) remains the gold standard for detection, provided that replicable material (i.e., nucleic acids) is present. However, PCR is rather time-consuming and expensive, and it requires expertise and laboratories. In a point-of-care situation, standard antigen detection is currently the choice; however, standard antigen detection often lacks desirable specificity and sensitivity. In such a situation, SPR-based sensor-like instruments can become a new paradigm: they provide higher specificity due to their single-particle detection capability and have the potential to ensure improved sensitivity. In the case of extracellular vesicles where no vesicle type defining replicable material (i.e., DNA, RNA) is available, and PCR is not applicable, SPR microscopy adds a new strategy to the analytical toolbox because it works directly with the NPs and the specific protein expression pattern presented on their surface. However, to complement or even outpace current methods, certain requirements must be met.

In the current research work, we not only aimed to report our new findings but also wanted to supply detailed SPR microscopy measurement protocols, aspiring to enhance the availability of this technique in the research fields dealing with nanoparticle characterization. In addition, we also aimed to verify whether the main analytical features of conventional SPR microscopy sensors remain persistent or even become improved after corrections of the tilted image plane (i.e., Scheimpflug correction). It was also important to define whether SPR microscopy sensors possess a sufficiently wide dynamic size range to analyze simultaneously biological objects belonging to different classes: for example, extracellular vesicles (nanoscale) and bacteria (microscale). Indeed, in the current research work, we demonstrated that the modified SPR microscopy sensor enables simultaneous detection of NPs that are significantly diverse in size. We detected simultaneously the binding of silica NPs (their refractive index is close to one of the biological NPs) with a four-times size difference. In [38], the binding of single micro-objects with a size of approximately 10 μm in diameter (lymphocytes) was observed. We, too, were able to detect the binding of HT29 cells (approximately 15 μm in diameter) to the gold surface using the SPR microscopy instrument (unpublished observation). Taken together, these findings indicate that biological objects that differ in size by at least two orders can be detected by employing the SPR microscopy instrument. The size resolution of the detectable NPs increases with the growth of the refractive index.

As SPR instruments are routinely employed to assess the formation of a layer of biomolecules on the sensor surface, it is straightforward to envision an instrument combining an opportunity to detect individual bio-NPs with an ability to monitor the formation of biomolecular layers. Findings reported in this work indicate that the SPR microscopy instrument can serve for both types of measurements. Acting this way, the SPR microscopy sensor can serve as a platform for the development of cell-based assays requiring simultaneous detection of soluble bio-molecules produced by cells (classical SPR features) and individual extracellular vesicles (SPR microscopy features).

The employment of an SPR microscope as a sensor instrument relies on its ability to automatize basic functionalities. We have presented an approach for automatically adjusting prism rotation and camera objective translation for the custom-constructed conventional SPR microscope. The prototype of this system was able to determine an optimal rotation angle and translation positions, with a quality comparable to a manual adjustment, autonomously. As the translation adjustment used the result of reference particle detection as a metric for calibration, this downstream task was directly considered in the optimization.

It has yet to be determined which concentrations of particles are required for reliable focus detection and by which amount the needed time for an automatic adjustment is reduced when using higher concentrations. It is also plausible that a calculation of the focus region is possible on raw images instead of preprocessed images, as focusing also affects non-particle signals: This means that even the disturbances visible in the raw images that occur due to defects in the film will appear sharper or blurrier, depending on the setting. Quantifying the sharpness of a raw image would consequently be further away from the actual downstream task of particle detection but has the potential to work without reference particles for calibration. Presumably, it could also be used as additional information to reduce the necessary positioning steps of the preprocessing-based focus determination.

## Figures and Tables

**Table 1:** Parameters and the assigned values for evaluating the presented focus determination methods.

Parameter	Value	Description
(*x*^(img)^, *y*^(img)^)	2592 × 1944	full image size
(*x*^(select)^, *y*^(select)^)	1600 × 300	size of tiles for selection
*n*_min_	10	minimum trace length
*d*_tol_	2	trace gap tolerance
*j*_min_	0.5	minimum Jaccard index for merging
*d*_*dil*_	32	dilation filter side length
*f*_cam_	15 fps	camera recording speed

## Data Availability

The data presented in this study are available upon request.

## Supplementary Materials

The following supporting information can be downloaded at: https://www.mdpi.com/article/10.3390/bios13040472/s1, Figure S1: (A) A gasket made of polydimethylsiloxane (PDMS) in order to prevent liquid leakage from the flow cell and aluminum stamps used for the gasket preparation, (B) wafer cleaving pliers (left) and a diamond cutter (right) instruments to cut gold slides in smaller pieces, which can be placed into the flow cell; Figure S2: (A) As the first step of SAM formation, functionalization of a gold sensor surface with Cys-protein A/G can be performed in the “coating chamber” overnight or in the flow cell under flow conditions in the SPR microscopy instrument. The next step is binding the anti-target antibody (in our case, anti-CD81) to the formed layer of Cys-protein A/G. We analyzed the efficiency of the anti-CD81 antibody binding to the layer of Cys-protein A/G formed in the “coating chamber” (green line) and in the flow cell of the SPR microscopy instrument (red line). (B) Intensity changes caused by the formation of the layer of anti-Spike protein SARS CoV2 antibody (HCAbs) onto the Cys-protein A/G layer. Reference [39] is cited in the Supplementary Material.

## Abbreviations

The following abbreviations are used in this manuscript:

bioNP	biological nanoparticle
CCD	charge-coupled device
CM	conditioned media
CMOS	complementary metal oxide semiconductor
EV	extracellular vesicle
FC	flow cytometry
MSC	human mesenchymal cell
NP	nanoparticle
SAM	self-assembling monolayer
SPR	surface plasmon resonance
VLP	virus-like particle
