# Supplementary material for: The Employment of the Surface Plasmon Resonance (SPR) Microscopy Sensor for the Detection of Individual Extracellular Vesicles and Non-Biological Nanoparticles
Source: Biosensors (Basel). 2023 Apr 12;13(4):472. doi: 10.3390/bios13040472 (PMC10136938; doi:10.3390/bios13040472)
Supplement: Supplementary file 1 [file biosensors-13-00472-s001.zip › biosensors-2288366-supplementary.pdf]

Supplementary Materials: The Employment of the Surface Plasmon Resonance (SPR) Microscopy Sensor for the Detection of Individual Extracellular Vesicles and Non-Biological Nanoparticles

Nour Sharar, Konstantin Wüstefeld, Rahat Morad Talukder, Julija Skolnik, Katharina Kaufmann, Bernd Giebel, Verena Börger, Friedrich Nolte, Carsten Watzl, Frank Weichert, Roland Hergenröder and Victoria Shpacovitch

1. Supplementary Materials

1.1. Cutting and Cleaning of Small Gold Coated Glass Plates—"Gold Sensors"— Before SPR Microscopy Experiments

The commercial glass slides mentioned in Section 2.1.1 in the main manuscript have to be cut into small pieces (plates) in order to fit the sizes of the flow cell (Figure 2A in the main manuscript) and a prism base (Figure 2B in the main manuscript). The cutting of the coated glass slides was performed using two types of instruments: a) a diamond cutter and b) wafer cleaving pliers produced by Micro to Nano, Netherlands (Cat# 52-002006; www.microtonano.com, accessed on 8 April 2023) (Figure S1B). These small plates (sizes 13×9 mm), received after cutting glass slides, are further named "gold sensors". Prior to functionalization, "gold sensors" have to be cleaned for approximately 5 min by freshly prepared "piranha solution" (1:3 ratio v/v the mixture of pre-chilled (at +4 °C) H_2O_2 and H_2SO_4; Caution! The acid should be added drop by drop to the desired volume of pre-chilled H_2O_2 since the reaction between these two compounds is exothermic and may lead to an explosion). Afterward, the gold sensors, which were treated this way, were washed with the flow of sterile and filtrated deionized water in order to remove the traces of "piranha solution" and dried under argon flow. Prepared gold sensors can be either immediately used for functionalization of their surface or stored under argon for up to one week at room temperature in a sealed box. During the mounting of a gold sensor inside the flow cell (Figure 2A in the main manuscript), a polydimethylsiloxane (PDMS) gasket is used to prevent the flow cell from leaking (Figure S1A). PDMS gasket was prepared using SYLGARD® 184 Kit produced by Sigma Aldrich (Cat# 761036-5EA). Custom-constructed aluminum stamps are necessary for gasket preparation.

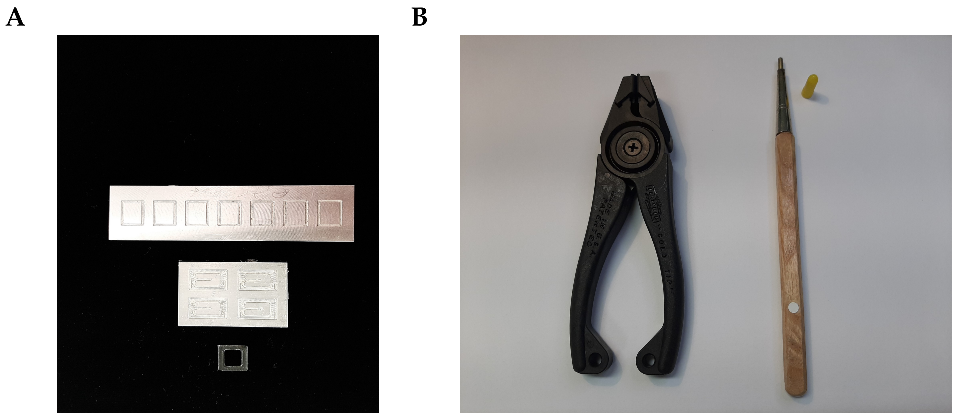

Figure S1. (**A**) A gasket made of polydimethylsiloxane (PDMS) in order to prevent liquid leakage from the flow cell and aluminum stamps used for the gasket preparation, (**B**) wafer cleaving pliers (left) and a diamond cutter (right) instruments to cut gold slides in smaller pieces, which can be placed into the flow cell.

1.2. Functionalization of the Sensor Surface for the Experiments With Inorganic Particles and Manual Processing of Their Binding Images

In the experiments with polystyrene 100 nm, 200 nm, 300 nm, and 400 nm in diameter or silica nanoparticles 200 nm and 800 nm in diameter, the following gold sensor surface functionalization was performed: sensor surface was covered with ″Nüscoflock″ in order to facilitate particle attachment to the gold sensor surface. ″Nüscoflock″ is a liquid containing 10% aluminum hydroxide chloride purchased from Dr. Nüsgen Chemie (Kamen, Germany). The liquid was filtrated before application on the gold sensor surface. The coating with ″Nüscoflock″ was performed outside the flow cell chamber of the SPR microscopy instrument. The coating was performed for at least 10 min at room temperature. After that, ″Nüscoflock″ was removed, and the gold sensor was washed with deionized water and dried under argon flow before it was placed in the flow cell of an SPR microscopy instrument. In earlier described studies [1,2], SPR microscopy measurements were performed using the GC2450 Prosilica camera (mentioned above) and the Streampix 3.0 or 5.0 (NorPix, Canada, www.norpix.com, accessed on 8 April 2023) camera operating software to record images of the NP binding to the gold sensor surface. The images recorded this way can also be analyzed manually employing the ImageJ software (http://rsb.info.nih.gov/ij). The processing of images using the ImageJ software is described in detail in [3,4]. During manual processing of the recorded images, signals caused by NP binding on the gold sensor surface were counted, and the magnitude of the intensity jump after a nanoparticle binding (″step signal″) was measured for each signal. A typical signal appears as a bright spot on the gray background image since a bound particle disturbs the evanescent field of the surface plasmon-polariton wave locally and, thus, locally supersedes reflectivity. The manually processed images also help to confirm a nanoparticle binding event due to the characteristic intensity changes ″step″ signal (Figure 6 in the main manuscript).

1.3. Nanoparticle Tracking Analysis (NTA) of Isolated EVs and Inorganic NPs

In order to control NPs characteristics, NTA measurements were performed. An LM10 instrument (NanoSight, UK; now a part of Malvern Pananalytical) equipped with a 405 nm laser was employed to carry out NTA measurements. Details of the measurement protocols are provided in [5]. Briefly, 0.02 µm filtrated water (for inorganic NPs) or an appropriate buffer (for EVs) was used to prepare and dilute the samples. Filtrated water and an appropriate buffer were routinely checked via NTA for contaminating NPs before the sample dilution. The minimum sample volume recommended for NTA analysis is 500 µL. A video of 90 s visualizing the Brownian motion of inorganic NPs or EVs was recorded using manufacturer software and further processed, and the concentration of visualized NPs or EVs, as well as their size, were determined. Measurements were performed at least in triplicates.

2. Supplementary Results

2.1. Monitoring the Formation of Self-Assembling Monolayers (SAMs) on the Gold Sensor Surface Employing a Modified SPR Microscopy Instrument

SPR microscopy instrument can be employed not only for visualization of the binding of individual bio-NPs such as extracellular vesicles or viruses, for example, but also for monitoring the formation of the layers of bio-molecules on the gold sensor surface. Moreover, the thickness of the formed layers may provide sufficient information for calculating the concentration of analyzed bio-molecules in the solution. Thus, the SPR microscopy sensor can be used not only as an instrument for quantification and sizing of individual bio-NPs but also as a classical SPR sensor to measure concentrations of target biomolecules in solution.

Certainly, this application requires the prior calibration of the instrument. The coating of a gold sensor chip with proteins (e.g., Cys-conjugated protein A/G, antibodies) can be performed not only in the flow cell of the SPR microscopy sensor but also using a custom-built ″coating chamber″ (Figure 5 in the main manuscript). This ″coating chamber″

was designed not only to minimize the time during which a gold sensor is placed in the SPR microscopy instrument but also to protect the glass side of the gold sensor from contact with bio-molecules. As it was revealed during our preliminary studies, the contact of immersion liquid with bio-molecules passively adsorbed on the glass side of the gold sensor can result in undesirable distortion of the resonance conditions (data not present). Thus, it was important to design the ″coating chamber″ in a way that prevents such type of contact. This issue was solved, as it is demonstrated in Figure 5 in the main manuscript. However, coating of the thin gold film with Cys-conjugated protein A/G in the ″coating chamber″ was performed with a higher concentration of the protein in comparison with coating performed under flow conditions. In the ″coating chamber″, a concentration of Cys-protein A/G employed for coating was 120 µg/mL. Afterward, it was important to verify whether coating of a gold sensor surface performed in the flow cell and in the ″coating chamber″ results in the formation of Cys-protein A/G layers similar in their efficiency to capture anti-target antibodies. Thus, gold sensors coated with Cys-protein A/G under flow conditions in SPR microscopy instrument and in the ″coating chamber″ were analyzed for their ability to capture anti-target antibodies under flow conditions. For such a proof-of-principle experiment, we used an anti-CD81 antibody. The results of this experiment are presented in Figure S2. These results demonstrate that the application of a ″coating chamber″ did not significantly affect the ability of the formed Cys-protein A/G layer to capture anti-target antibodies. For us, it was also essential to check whether the Cys-protein A/G layer formed onto a gold sensor chip surface may capture modified antibodies such as heavy-chain only antibodies (HCAbs). For our experiments, the anti-Spike protein of the virus SARS-CoV2 HCAbs was used (RBD-specific HCAb). These HCAbs were created by the working team of Prof. Dr. Nolte. The results of this experiment are presented in Figure S2. Thus, it is demonstrated that the Cys-protein A/G layer formed on the gold sensor chip surface can also bind HCAbs.

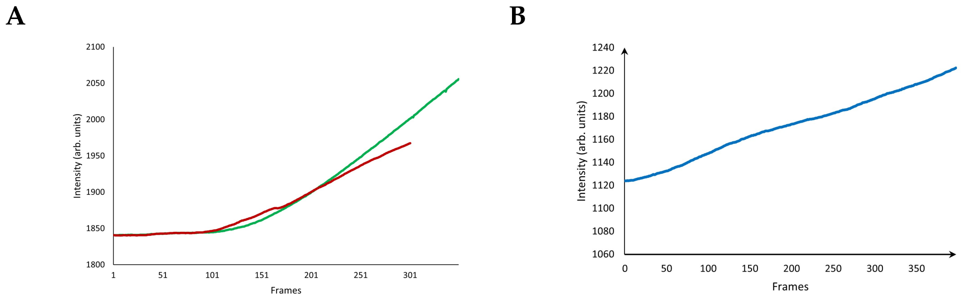

Figure S2. (**A**) As the first step of SAM formation, functionalization of a gold sensor surface with Cys-protein A/G can be performed in the "coating chamber″ overnight or in the flow cell under flow conditions in the SPR microscopy instrument. The next step is binding the anti-target antibody (in our case, anti-CD81) to the formed layer of Cys-protein A/G. We analyzed the efficiency of the anti-CD81 antibody binding to the layer of Cys-protein A/G formed in the "coating chamber″ (green line) and in the flow cell of the SPR microscopy instrument (red line). (**B**) Intensity changes caused by the formation of the layer of anti-Spike protein SARS CoV2 antibody (HCAbs) onto the Cys-protein A/G layer.

1. Shpacovitch, V.; Temchura, V.; Matrosovich, M.; Hamacher, J.; Skolnik, J.; Libuschewski, P.; Siedhoff, D.; Weichert, F.; Marwedel, P.; Müller, H.; et al. Application of surface plasmon resonance imaging technique for the detection of single spherical biological submicrometer particles. *Anal. Biochem.* **2015**, *486*, 62–69.
2. Shpacovitch, V.; Sidorenko, I.; Lenssen, J.E.; Temchura, V.; Weichert, F.; Müller, H.; Überla, K.; Zybin, A.; Schramm, A.; Hergenröder, R. Application of the PAMONO-sensor for quantification of microvesicles and determination of nano-particle size distribution. *Sensors* **2017**, *17*, 244.

3. Zybin, A.; Kuritsyn, Y.A.; Gurevich, E.L.; Temchura, V.V.; Überla, K.; Niemax, K. Real-time detection of single immobilized nanoparticles by surface plasmon resonance imaging. *Plasmonics* **2010**, *5*, 31–35.
4. Gurevich, E.; Temchura, V.; Überla, K.; Zybin, A. Analytical features of particle counting sensor based on plasmon assisted microscopy of nano objects. *Sens. Actuators B Chem.* **2011**, *160*, 1210–1215.
5. Usfoor, Z.; Kaufmann, K.; Rakib, A.S.H.; Hergenröder, R.; Shpacovitch, V. Features of Sizing and Enumeration of Silica and Polystyrene Nanoparticles by Nanoparticle Tracking Analysis (NTA). *Sensors* **2020**, *20*, 6611.
